# A Prospective Study on Red Blood Cell Transfusion Related Hyperkalemia in Critically Ill Patients

**DOI:** 10.14740/jocmr2123w

**Published:** 2015-04-08

**Authors:** Shahzad Raza, Mahadi Ali Baig, Christopher Chang, Ridhima Dabas, Mallika Akhtar, Areej Khan, Krishna Nemani, Rahima Alani, Omran Majumder, Natalya Gazizova, Shaluk Biswas, Priyeshkumar Patel, Jaffar A. Al-Hilli, Yasar Shad, Barbara J. Berger, Mohammad Zaman

**Affiliations:** a Department of Internal Medicine, Brookdale University Hospital & Medical Center, 1 Brookdale Plaza, Brooklyn, New York, NY 11212, USA; b University of Missouri Columbia, Ellis Fischel Cancer Center, Columbia, Missouri, MO 65212, USA; c Albert Einstein School of Medicine, Montefiore Medical Center, 111 East 210th Street, Bronx, NY 10467, USA

**Keywords:** Hyperkalemia, Red cell storage, Packed red blood cell transfusion, Potassium

## Abstract

**Background:**

Transfusion-associated hyperkalemic cardiac arrest is a serious complication in patients receiving packed red blood cell (PRBC) transfusions. Mortality from hyperkalemia increases with large volumes of PRBC transfusion, increased rate of transfusion, and the use of stored PRBCs. Theoretically, hyperkalemia may be complicated by low cardiac output, acidosis, hyperglycemia, hypocalcemia, and hypothermia. In this study, we focus on transfusion-related hyperkalemia involving only medical intensive care unit (MICU) patients.

**Method:**

This prospective observational study focuses on PRBC transfusions among MICU patients greater than 18 years of age. Factors considered during each transfusion included patient’s diagnosis, indication for transfusion, medical co-morbidities, acid-base disorders, K^+^ levels before and after each PRBC transfusion, age of stored blood, volume and rate of transfusion, and other adverse events. We used Pearson correlation and multivariate analysis for each factor listed above and performed a logistic regression analysis.

**Results:**

Between June 2011 and December 2011, 125 patients received a total of 160 units of PRBCs. Median age was 63 years (22 - 92 years). Seventy-one (57%) were females. Sixty-three patients (50%) had metabolic acidosis, 75 (60%) had acute renal failure (ARF), and 12 (10%) had end-stage renal disease (ESRD). Indications for transfusion included septic shock (n = 65, 52%), acute blood loss (n = 25, 20%), non-ST elevation myocardial infarction (NSTEMI) (n = 25, 20%) and preparation for procedures (n = 14, 11%). Baseline K^+^ value was 3.9 ± 1.1 mEq/L compared to 4.3 ± 1.2 mEq/L post-transfusion respectively (P = 0.9). During this study period, 4% of patients developed hyperkalemia (K^+^ 5.5 mEq/L or above). The mean change of serum potassium in patients receiving transfusion ≥ 12 days old blood was 4.1 ± 0.4 mEq/L compared to 4.8 ± 0.3 mEq/L (mean ± SD) in patients receiving blood 12 days or less old. Sixty-two patients (77.5%) that were transfused stored blood (for more than 12 days) had increased serum K^+^; eight (17.7%) patients received blood that was stored for less than 12 days. In both univariate (P = 0.02) and multivariate (P = 0.04) analysis, findings showed that among all factors, transfusion of stored blood was the only factor that affected serum potassium levels (95% CI: 0.32 - 0.91). No difference was found between central and peripheral intravenous access (P = 0.12), acidosis (P = 0.12), ARF (P = 0.6), ESRD (P = 0.5), and multiple transfusions (P = 0.09). One subject developed a sustained cardiac arrest after developing severe hyperkalemia (K^+^ = 9.0) following transfusion of seven units of PRBCs. Multivariate logistic regression showed linear correlation between duration of stored blood and serum K^+^ (R^2^ = 0.889).

**Conclusion:**

This study assesses factors that affect K^+^ in patients admitted to MICU. Results from the study show that rise in serum K^+^ level is more pronounced in patients who receive stored blood (> 12 days). Future studies should focus on the use of altered storage solution, inclusion of potassium absorption filters during transfusion and cautious use of blood warmer in patients requiring massive blood transfusions.

## Introduction

Transfusion of stored red blood cells (RBCs) is associated with a wide range of complications that include circulatory overload, bradykinin-mediated hypotension, allergic reactions, coagulopathies, acute lung injury, infections, and death [1]. Hyperkalemia is a common complication in transfusion of stored blood. The supernatant of stored RBCs usually contains more than 60 mEq/L of potassium [2]. Potassium in stored blood increases due to decrease in ATP production and leakage of potassium into the supernatant. The initial high levels of potassium in stored blood predispose to post-transfusion hyperkalemia.

Cardiac arrest has been commonly reported in transfusion-associated hyperkalemia. Most cardiac arrests were reported in children and adult patients requiring massive blood transfusions [3, 4]. Hypokalemia was rarely reported as a transfusion-associated complication [5]. Recent research has focused on “stored blood” with growing concern that the age of an RBC unit (even with the current accepted shelf life of 42 days) may affect its safety profile. A large multicenter clinical trial is underway to determine the effects of using standard storage-age RBCs as compared to fresh blood (8 days old) (Age of Blood Evaluation (ABLE) trial, ISRCTN44878718) [1].

Pre-washing of RBCs is an essential practice for reducing potassium load in irradiated PRBCs [6, 7]. Due to the limited availability of time for preparing washed PRBCs in urgent situations, trauma patients usually receive unwashed RBCs that lead to increased risk for post-transfusion hyperkalemia [7].

Although RBC washing is useful in reducing the potassium load during transfusion, the effectiveness of this preventative technique is unknown in patients with medical co-morbidities such as metabolic acidosis, acute renal failure (ARF), end-stage renal disease (ESRD), rhabdomyolysis, hypothermia and shock. In this study, we evaluate the medical morbidity factors and transfusion factors that can contribute to changes in serum potassium levels among critically ill patients.

### Aims and objectives

Primary objective was to assess the statistical significance of the change in serum potassium levels among critically ill patients before and after each unit of PRBC transfusion. Secondary objectives include factors that can potentially cause rise in serum potassium levels in these patients.

## Patients and Methods

### Study design

This is a prospective observational study conducted at Brookdale Hospital from June 2011 to December 2011 that was approved by The Investigational Review Board of Brookdale University Hospital and Medical Center, New York, USA. The study included all consecutive series of patients, greater than 18 years of age, who were admitted to the medical intensive care unit (MICU) and required blood transfusions. Subjects excluded from the study were MICU patients receiving fresh frozen plasmas, platelets, cryoprecipitates and multiple blood products simultaneously. Patients with ESRD receiving PRBC transfusions during dialysis were also excluded.

We collected data on demographics admitting diagnosis, acid-base disorders and basic metabolic panel prior to each RBC transfusion. Additional information on date of RBC donation, irradiated RBCs, washed PRBC volume and RBC volume (mL) were collected. We repeated basic metabolic panels 4 h from the time of initiation of blood transfusion. Adverse events were recorded during transfusion and 4 h after transfusion. Our criteria for defining transfusion-associated hyperkalemia included laboratory evidence of K^+^ levels 5.5 mEq/L or above.

Data on factors that can potentially affect serum K^+^ concentration were collected and analyzed using SPSS version 15. In addition to medical morbidities, additional data were collected on use of diuretics, vasodilators (angiotensin converting enzyme inhibitors or angiotensin receptor blockers), spironolactones and potassium supplements in intravenous fluid. The primary outcome was the development of hyperkalemia (plasma potassium level greater than or equal to 5.5 mEq/L) after a blood transfusion and to assess the change in serum potassium level. Secondary analysis included correlation between the numbers of factors, which can potentially cause rise in serum potassium levels.

### Statistics

SPSS 15.0 (SPSS, Chicago, IL) was used for all data analyses. The baseline data of patients are assessed using frequency distributions and univariate descriptive statistics including measures of central tendency and dispersion. All variables that could potentially affect changes in potassium levels were included in analysis via Pearson correlation. Finally, variables associated with a P value of less than 0.05 in univariate analysis were included in the logistic regression model for multivariate analysis. Logistic regression analysis was performed separately to assess factors independently associated with the development of hyperkalemia.

## Results

One hundred twenty-five patients received 160 units of PRBC infusions from June 2011 to December 2011. Table 1 summarizes the characteristics of patient population admitted to MICU and required PRBC transfusion. The median age of the study group was 63 years (range 22 - 92 years). Seventy-one (57%) were female and 54 (43%) were male. Indications for blood transfusions include septic shock (n = 61), non-ST elevation myocardial infarction (NSTEMI) (n = 25), acute blood loss due to gastrointestinal bleeding (n = 15), disseminated intravascular coagulation (n = 9) and vaginal bleed (n = 1). Fourteen patients received transfusion prior to invasive procedure which includes sigmoidoscopy (n = 5), tracheotomy (n = 6), liver biopsy (n = 1) and debridement of decubitus ulcers (n = 2).

**Table 1:** Patient Characteristics

Gender	N = 125	%
Female	71	56
Male	54	43.2
Age	63 (median)	22 - 92 years
Indication for PRBC transfusion		
Acute blood loss	25	16.6
Coronary artery disease with NSTEMI	25	16.6
Septic shock	61	49
Invasive procedure	14	11.2
No. of PRBC transfusions		
1 unit	104	83.2
2 units	11	8.8
3 units	9	7.2
7 units	1	0.8
Intravenous access		
Central line	72	57.6
Peripheral line	53	42.4
Age of blood	15 days	2 - 36 days
Medications		
ACE /ARB inhibitors	21	16.8
Spironolactone	8	6.4
Insulin	38	30.4
Diuretics	66	52.8
Morbidities		
Acidosis (pH < 7.3)	63	50.4
Acute renal failure	75	60
End-stage renal disease	12	9.6

For data analysis, patient received multiple blood transfusions at a given period, 4 h K^+^ level from the initiation of first transfusion or the time of basic metabolic panel drawn after first transfusion was used for analysis. In all cases, basic metabolic panel was drawn immediately after transfusion. However, K^+^ level was also measured at the end of multiple blood transfusion to assess the change in potassium at the given period.

For the primary outcome, the mean change of serum K^+^ was 3.9 ± 1.1 mEq/L (mean ± SD) compared to 4.3 ± 1.2 mEq/L 4 h post-transfusion for 160 transfused units of PRBC volume. Four percent of patients (n = 5) had post-transfusion hyperkalemia whereas 1.6% (n = 2) developed hypokalemia after transfusion. We noticed higher serum K^+^ value in 73 patients (58.4%) compared to 52 patients (41.6%). The change of serum potassium was statistically not significant (P = 0.1). Table 2 describes the association of each potential factors and development of hyperkalemia in univariate and multivariate analysis.

**Table 2:** Univariate and Multivariate Analysis of Factors Affecting K^+^ Value

Factors affecting K^+^ value	Univariate	95% confidence interval	Multivariate	95% confidence interval
Change in K^+^ value pre- and post-transfusion	0.2	0.58 - 1.93	0.3	0.31 - 2.88
Acidosis	0.1	0.22 - 1.67	0.2	0.62 - 3.02
Acute renal failure	0.64	0.28 - 3.33	0.55	0.47 - 2.17
End-stage renal disease	0.52	0.26 - 2.12	0.97	0.91 - 4.92
No. of transfusions	0.11	0.12 - 1.83	0.32	0.48 - 2.25
Age of RBC blood	0.02	0.32 - 0.91	0.04	0.14 - 0.91
K^+^ infusion via central access vs. peripheral access	0.43	0.75 - 4.17	0.82	0.76 - 3.93

**Figure 1:**
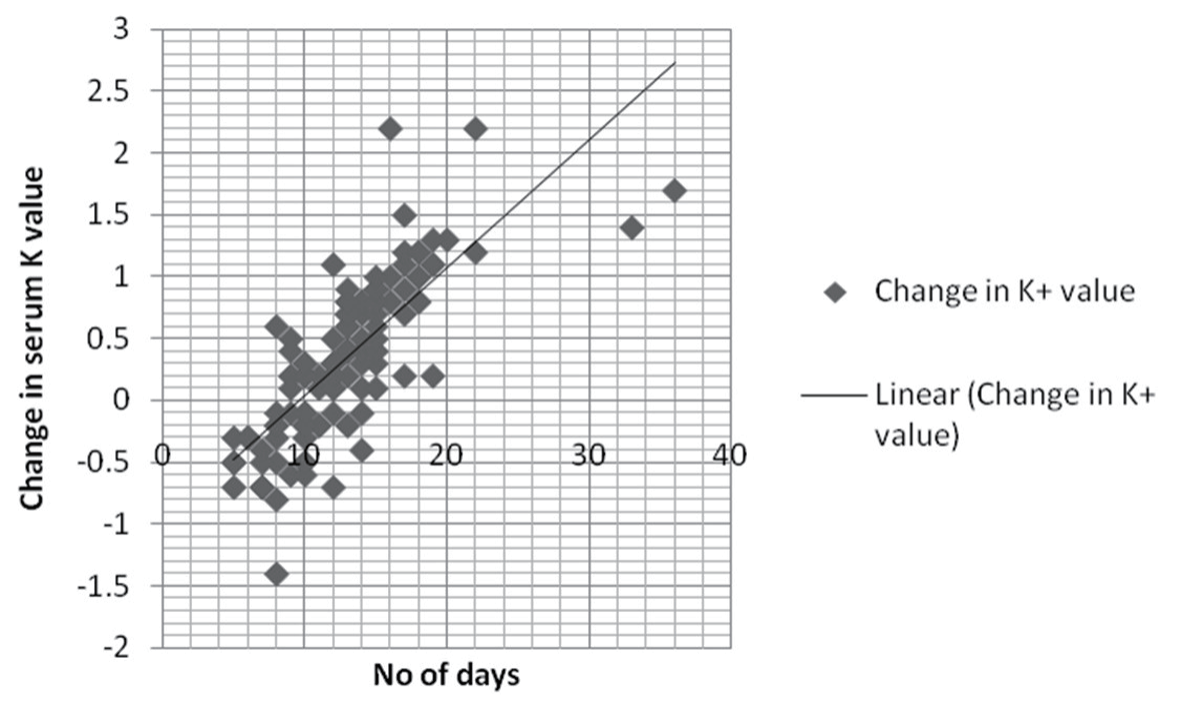
Linear correlation between number of days and serum potassium (K^+^) level.

We analyzed multiple patient factors that can potentially affect serum K^+^ level like acid-base disorder (pH < 7.3 or > 7.5) (P = 0.17), ARF (creatinine 1.3 or above) (P = 0.61), ESRD (P = 0.58) and medications (P = 0.44). Mean age of stored blood defined from the time of donation to the time of administration was found to be 12 days (range: 2 - 36 days). Twelve days older blood was used in 64% of patients (n = 80) during first transfusion compared to 45 patients (36%) who received blood of less than 12 days of older. The mean change of serum potassium in patients receiving transfusion ≥ 12 days old blood was 4.1 ± 0.4 mEq/L compared to 4.8 ± 0.3 mEq/L (mean ± SD) in patients receiving blood 12 days or less old. Sixty-two patients (77.5%) had increased change of serum K^+^ value in patients with > 12 days old blood transfusion compared to 17.7% (n = 8) patients (P = 0.02). In multivariate logistic regression model, the use of stored PRBC for transfusion was the only factor that affects the increased change in serum K^+^ value (P = 0.04). The change of K^+^ value was independent of acidosis, medications, renal failure, central line versus peripheral line access, and the rate and volume of blood transfusion. Linear regression multivariate analysis for the relationship of transfused stored blood for more than 12 days and the rise in K^+^ level was found to be 0. 889. Figure 1 describes the linear correlation of change in serum potassium and stored blood.

In our study patients, one patient required massive PRBC transfusion due to severe gastrointestinal bleed. Seven units of transfusions were used via pressure bags. However, the PRBC used in the patient were unwashed which could explain the increase in serum potassion from 5.3 mEq/L to 6.3 mEq/L. One death occurred from cardiac arrest after receiving seven units of PRBCs (9 mEq/L).

## Discussion

Hyperkalemia is one of the most serious electrolyte disturbances that can lead to lethal cardiac arrhythmias. Smith and colleagues reported 16 patients including 11 adults and five pediatric patients who had cardiac arrests with hyperkalemia during PRBC transfusion with a survival rate of 12.5% [8]. These findings are reported mostly in surgical literature in settings of large-volume transfusions with other blood products. Therefore, some uncertainty remains to whether use of packed PRBCs is the sole factor which can cause hyperkalemia [4].

Laboratory and observational studies have raised the possibility of prolonged RBC storage that may adversely affect clinical outcomes. In our study, we found that the use of stored PRBC infusions for more than 12 days may lead to elevated K^+^. However, the magnitude of change in K^+^ by itself is non-significant which suggests that present techniques of irradiated washed RBCs to some extent have decreased the complications of hyperkalemia. Stored PRBCs should be used with caution in patients with previously elevated serum K^+^ levels.

In previous reports [3-5], volume of transfusion and rate of PRBCs were factors attributed to rise in K^+^ levels. However, most of these patients received unwashed PRBC transfusions in emergent circumstances that lead to hyperkalemia [3-7]. The citrate-phosphate-dextrose-adenine (CPDA-1) and citrate-phosphate-dextrose (CPD) storage RBCs have shown a 10-fold rise in K^+^ value through day 1 to day 42 due to decreased RBC survival, increased fragility, decreased viability, and increased deformability of RBCs as well as the release of a number of substances resulting in adverse systemic responses as fever, cellular injury, alterations in regional and global blood flow, and organ dysfunction [9-13].

This is the first study assessing factors that may cause hyperkalemia in critically ill patients. Compared to pediatric and trauma patients, critically ill patients usually receive leuko-reduced and washed PRBCs. Therefore, the change in serum K^+^ value was never addressed in these patients despite of all these factors. Another important aspect in this study was to measure the K^+^ level immediately after PRBC transfusion. This strategy was employed based on the study by Dani et al [14], where authors have implicated measuring serum K^+^ value immediately after transfusion has provided the accurate estimate of K^+^ level in their patient population.

In addition to our findings, few clinical trials are testing the hypothesis of increased mortality with use of stored RBCs. The ABLE study is comparing the transfusion of leuko-reduced RBCs stored for 7 days or less (fresh arm) compared to standard-issue RBCs stored, on average, 15 - 20 days (control arm) for 90-day all-cause mortality and reduced morbidity in critically ill adults [9].

More recently, RBC irradiation has shown to damage the RBC membrane that increases membrane permeability and leakage of potassium into the extracellular space that increases extracellular K^+^ concentration [15]. The washing of RBCs is then performed to reduce K^+^ content, which has been routinely done prior to blood transfusion in medically ill patients in the United States [15]. Therefore, use of washed PRBCs is considered the ideal RBC product in critically ill medical patients. Since washing of PRBCs requires considerable time, patients who require massive transfusions are transfused unwashed PRBCs. As a result, there is a high theoretical risk of hyperkalemia in these patients.

In the past, majority of these studies were focused on pediatric and adult patients requiring multiple transfusions. Our study demonstrates that stored blood K^+^ load can be attributed to increased serum K^+^ value post-transfusion. This factor was independent on rate and volume of PRBC infusions and medical morbidities. Theoretically, transfusion of larger volumes at increased rate may have the potential to cause a greater predisposition to hyperkalemia. However, in our study, massive transfusion-associated hyperkalemia was noticed only in one patient. The effect of serum potassium levels with large volumes of PRBC transfusion should be closely studied in patients requiring rapid transfusions.

The availability of storage raises the question of how long blood products can and should be stored and how long they are safe and effective and there are no prospective randomized evidence that old RBCs increase mortality despite of theoretical risk [16, 17]. Few studies are on-going to assess the safety of storage of PRBCs. The ABLE study in critically ill patients and the ARIPI study (NCT00326924) in neonates are assessing the safety of 7 days older storage PRBCs. The “Red Cell Storage Duration and Outcomes in Cardiac Surgery” (NCT00458783) is a single-center RCT studying the safety of 14 days or older PRBCs and “The Red Cell Storage Age Study (NCT00991341) is evaluating the safety of 10 days or older blood. The results of these studies will help to identify the safety cut-off of storage PRBCs [9].

### Conclusion

This is the first prospective study that assesses the change in serum K^+^ levels in critically ill patients. The risk of hyperkalemia is minimal in our patient population due to the use of washed PRBCs. However, our study questions the safety of transfusion of PRBCs stored for greater than 12 days. Determining the optimal duration of RBC storage maximizes safety of transfusion. Future studies should focus on the use of altered storage solution, inclusion of potassium absorption filters during transfusion and cautious use of warmer blood in the hopes of minimizing its clinical consequences through the development of better storage methods [17].
